# Supplementary material for: Effect of AGTR1 A1166C genetic polymorphism on coronary artery lesions and mortality in patients with acute myocardial infarction
Source: PLoS One. 2024 Apr 18;19(4):e0300273. doi: 10.1371/journal.pone.0300273 (PMC11026145; doi:10.1371/journal.pone.0300273)
Supplement: S1 File — (DOCX) [file pone.0300273.s001.docx]

**Supplementary material**. PCR primers, components, and conditions for *AGTR1* A1166C genotyping

**S1 Table. PCR Primers.**

| **Primer** | **Genetic sequence** | **Amplified length (bp)** | **Primer ratio** | **Tm**  **(^0^C)** |
| --- | --- | --- | --- | --- |
| AGTR1-F2 | 5’- GCCATGCCTATCACCATTTG -3’ | Control: 544  Allele A:342 | 1 | 54.3 |
| AGTR1-R2 | 5’- AAAGCAGGCTAGGGAGATTG -3’ |  | 1 | 54.6 |
| AGTR1-1166A-R2 | 5’- TTCAATTCTGAAAAGTAGCTCA**T** -3’ |  | 1 | 50.3 |
| AGTR1-F2 | 5’- GCCATGCCTATCACCATTTG -3’ | Control: 544  Allele C: 342 | 1 | 54.3 |
| AGTR1-R2 | 5’- AAAGCAGGCTAGGGAGATTG -3’ |  | 1 | 54.6 |
| AGTR1-1166C-R2 | 5’-TTCAATTCTGAAAAGTAGCTCA**G** -3’ |  | 1 | 51.0 |

**S2 Table. PCR components.**

| Component | Volume (µL) |
| --- | --- |
| 3 primers | 1.5 (0.5 each) |
| PCR buffer 10X | 1.5 |
| TaKaRa Taq^TM^ HotStart Polymerase (Takara BiBio INC., Japan) | 0.1 |
| dNTP 2.5 mM | 1.5 |
| gDNA (20-50 ng/µL) | 2 |
| De-ionised water | 8.4 |
| Total | **15** |

**S3 Table. PCR conditions.**

| Step | Temperature (^0^C) | Time |
| --- | --- | --- |
| Initialization | 98 | 3 minutes |
| 40 cycles  - Denaturation  - Annealing  - Extension | 98  56  72 | 20 seconds  20 seconds  30 seconds |
| Elongation | 72 | 2 minutes |
