# Supplementary material for: Effect of AGTR1 A1166C genetic polymorphism on coronary artery lesions and mortality in patients with acute myocardial infarction
Source: PLoS One. 2024 Apr 18;19(4):e0300273. doi: 10.1371/journal.pone.0300273 (PMC11026145; doi:10.1371/journal.pone.0300273)
Supplement: Attachment — Submitted filename: PONE-D-23-38605_reviewer.pdf [file pone.0300273.s004.pdf]

PLOS ONE

Effect of AGTR1 A1166C Genetic Polymorphism on Coronary Artery Lesions and Mortality in Patients with Acute Myocardial Infarction

--Manuscript Draft--

**Manuscript Number:**	PONE-D-23-38605
**Article Type:**	Research Article
**Full Title:**	Effect of AGTR1 A1166C Genetic Polymorphism on Coronary Artery Lesions and Mortality in Patients with Acute Myocardial Infarction
**Short Title:**	Role of AGTR1 A1166C in Acute Myocardial Infarction
**Corresponding Author:**	Minh Duc Do, MD, Ph.D<br>University Of Medicine and Pharmacy at Ho Chi Minh City<br>Ho Chi Minh City, Please Select VIET NAM
**Keywords:**	AGTR1 A1166C; genetic polymorphism; coronary artery lesion; mortality; acute myocardial infarction
**Abstract:**	Background<br><br>The pathogenesis and prognosis of patients with acute myocardial infarction (AMI) may be influenced by both genetic and environmental factors. Findings on the relationship of polymorphisms in genes encoding the renin-angiotensin-aldosterone system with coronary artery lesions and mortality in AMI patients are inconsistent. The aim of this study was to determine whether the AGTR1 A1166C genetic polymorphism affects coronary artery lesions and 1-year mortality in post-AMI patients.<br><br>Methods<br><br>Patients with their first AMI admitted to Cho Ray Hospital, Vietnam, from January 2020 to August 2021 were enrolled in this prospective clinical study. All participants underwent invasive coronary angiography and were identified as having the genotypes of AGTR1 A1166C by way of a polymerase chain reaction method. All patients were followed up for all-cause mortality 12 months after AMI. The association of the AGTR1 A1166C polymorphism with coronary artery lesions and 1-year mortality were evaluated using logistic regression and Cox regression analysis, respectively.<br><br>Results<br><br>531 AMI patients were recruited. The mean age was 63.9 ± 11.6 years, and 71.6% of the patients were male. There were no significant differences in the location and number of diseased coronary artery branches between the AA and AC+CC genotypes. The AC and CC genotypes were independently associated with ≥ 90% diameter stenosis of the left anterior descending (LAD) artery (odds ratio = 1.940; 95% confidence interval (CI): 1.059 - 3.552, p = 0.032). The difference in the 1-year all-cause mortality rate between patients with the AC and CC genotypes versus those with the AA genotype was not statistically significant (hazard ratio = 1.000, 95% CI: 0.429–2.328, p = 1.000).<br><br>Conclusions<br><br>The AGTR1 A1166C genetic polymorphism is associated with very severe luminal stenosis of the LAD, but not with mortality in AMI patients.
**Order of Authors:**	Duy Cong Tran
	Linh Hoang Gia Le
	Truc Thanh Thai
	Sy Van Hoang
	Minh Duc Do, MD, Ph.D

Powered by Editorial Manager® and ProduXion Manager® from Aries Systems Corporation

	Binh Quang Truong
**Additional Information:**	
**Question**	**Response**
**Financial Disclosure**<br><br>Enter a financial disclosure statement that describes the sources of funding for the work included in this submission. Review the submission guidelines for detailed requirements. View published research articles from *PLOS ONE* for specific examples.<br><br>This statement is required for submission and **will appear in the published article** if the submission is accepted. Please make sure it is accurate.<br><br>**Funded studies**<br>Enter a statement with the following details:<br>• Initials of the authors who received each award<br>• Grant numbers awarded to each author<br>• The full name of each funder<br>• URL of each funder website<br>• Did the sponsors or funders play any role in the study design, data collection and analysis, decision to publish, or preparation of the manuscript?<br><br>Did you receive funding for this work?	Yes
Please add funding details.<br>as follow-up to "**Financial Disclosure**<br><br>Enter a financial disclosure statement that describes the sources of funding for the work included in this submission. Review the submission guidelines for detailed requirements. View published research articles from *PLOS ONE* for specific examples.<br><br>This statement is required for submission and **will appear in the published article** if the submission is accepted. Please make sure it is accurate.	University of Medicine and Pharmacy at Ho Chi Minh City.

**Funded studies**<br>Enter a statement with the following details:<br>• Initials of the authors who received each award<br>• Grant numbers awarded to each author<br>• The full name of each funder<br>• URL of each funder website<br>• Did the sponsors or funders play any role in the study design, data collection and analysis, decision to publish, or preparation of the manuscript?<br><br>Did you receive funding for this work?"	
Please select the country of your main research funder (please select carefully as in some cases this is used in fee calculation).<br>as follow-up to "**Financial Disclosure**<br><br>Enter a financial disclosure statement that describes the sources of funding for the work included in this submission. Review the submission guidelines for detailed requirements. View published research articles from *PLOS ONE* for specific examples.<br><br>This statement is required for submission and **will appear in the published article** if the submission is accepted. Please make sure it is accurate.<br><br>**Funded studies**<br>Enter a statement with the following details:<br>• Initials of the authors who received each award<br>• Grant numbers awarded to each author<br>• The full name of each funder<br>• URL of each funder website<br>• Did the sponsors or funders play any role in the study design, data collection and analysis, decision to publish, or preparation of the manuscript?<br><br>Did you receive funding for this work?"	VIET NAM - VN
**Competing Interests**	No

Powered by Editorial Manager® and ProduXion Manager® from Aries Systems Corporation

Use the instructions below to enter a competing interest statement for this submission. On behalf of all authors, disclose any competing interests that could be perceived to bias this work—acknowledging all financial support and any other relevant financial or non-financial competing interests.<br><br>This statement is required for submission and **will appear in the published article** if the submission is accepted. Please make sure it is accurate and that any funding sources listed in your Funding Information later in the submission form are also declared in your Financial Disclosure statement.<br><br>View published research articles from *PLOS ONE* for specific examples.<br><br>**NO authors have competing interests**<br><br>Enter: *The authors have declared that no competing interests exist.*<br><br>**Authors with competing interests**<br><br>Enter competing interest details beginning with this statement:<br><br>*I have read the journal's policy and the authors of this manuscript have the following competing interests: [insert competing interests here]*<br><br>* typeset	
**Ethics Statement**<br><br>Enter an ethics statement for this submission. This statement is required if the study involved:<br><br>• Human participants<br>• Human specimens or tissue<br>• Vertebrate animals or cephalopods<br>• Vertebrate embryos or tissues	The study was approved by the local ethics committee of University of Medicine and Pharmacy at Ho Chi Minh City (Protocol No. 550/UMP-BOARD). All patients gave their informed consent to participate in this study

Powered by Editorial Manager® and ProduXion Manager® from Aries Systems Corporation

• Field research<br><br>Write "N/A" if the submission does not require an ethics statement.<br><br>General guidance is provided below. Consult the submission guidelines for detailed instructions. **Make sure that all information entered here is included in the Methods section of the manuscript.**	

**Format for specific study types**<br><br>**Human Subject Research (involving human participants and/or tissue)**<br>• Give the name of the institutional review board or ethics committee that approved the study<br>• Include the approval number and/or a statement indicating approval of this research<br>• Indicate the form of consent obtained (written/oral) or the reason that consent was not obtained (e.g. the data were analyzed anonymously)<br><br>**Animal Research (involving vertebrate animals, embryos or tissues)**<br>• Provide the name of the Institutional Animal Care and Use Committee (IACUC) or other relevant ethics board that reviewed the study protocol, and indicate whether they approved this research or granted a formal waiver of ethical approval<br>• Include an approval number if one was obtained<br>• If the study involved *non-human primates*, add *additional details* about animal welfare and steps taken to ameliorate suffering<br>• If anesthesia, euthanasia, or any kind of animal sacrifice is part of the study, include briefly which substances and/or methods were applied<br><br>**Field Research**<br><br>Include the following details if this study involves the collection of plant, animal, or other materials from a natural setting:<br>• Field permit number<br>• Name of the institution or relevant body that granted permission	
**Data Availability**<br><br>Authors are required to make all data underlying the findings described fully available, without restriction, and from the time of publication. PLOS allows rare exceptions to address legal and ethical concerns. See the PLOS Data Policy and FAQ for detailed information.	Yes - all data are fully available without restriction

A Data Availability Statement describing where the data can be found is required at submission. Your answers to this question constitute the Data Availability Statement and **will be published in the article**, if accepted.<br><br>**Important:** Stating 'data available on request from the author' is not sufficient. If your data are only available upon request, select 'No' for the first question and explain your exceptional situation in the text box.<br><br>Do the authors confirm that all data underlying the findings described in their manuscript are fully available without restriction?	
**Describe where the data may be found in full sentences. If you are copying our sample text, replace any instances of XXX with the appropriate details.**<br><br>• If the data are **held or will be held in a public repository**, include URLs, accession numbers or DOIs. If this information will only be available after acceptance, indicate this by ticking the box below. For example: *All XXX files are available from the XXX database (accession number(s) XXX, XXX.).*<br>• If the data are all contained **within the manuscript and/or Supporting Information files**, enter the following: *All relevant data are within the manuscript and its Supporting Information files.*<br>• If neither of these applies but you are able to provide **details of access elsewhere**, with or without limitations, please do so. For example:<br><br>*Data cannot be shared publicly because of [XXX]. Data are available from the XXX Institutional Data Access / Ethics Committee (contact via XXX) for researchers who meet the criteria for access to confidential data.*<br><br>*The data underlying the results presented in the study are available from (include the name of the third party*	All relevant data are within the manuscript and its Supporting Information files

and contact information or URL).<br>• This text is appropriate if the data are owned by a third party and authors do not have permission to share the data.<br><br>* typeset	
Additional data availability information:	

Manuscript
Click here to access/download;Manuscript;Effect of AGTR1_PO.docx

Title

Effect of *AGTR1* A1166C Genetic Polymorphism on Coronary Artery Lesions and Mortality in Patients with Acute Myocardial Infarction

Authors:

Duy Cong Tran[1,2,3], Linh Hoang Gia Le[4], Truc Thanh Thai[5], Sy Van Hoang[1,2], Minh Duc Do[4*], Binh Quang Truong[1,3*]

Affiliations:

[1]Department of Internal Medicine, University of Medicine and Pharmacy at Ho Chi Minh City, 700000 Ho Chi Minh City, Vietnam

[2]Department of Cardiology, Cho Ray Hospital, 700000 Ho Chi Minh City, Vietnam

[3]Cardiovascular Center, University Medical Center Ho Chi Minh City, 700000 Ho Chi Minh City, Vietnam

[4]Center for Molecular Biomedicine, University of Medicine and Pharmacy at Ho Chi Minh City, 700000 Ho Chi Minh City, Vietnam

[5]Faculty of Public Health, University of Medicine and Pharmacy at Ho Chi Minh City, 700000 Ho Chi Minh City, Vietnam

***Correspondence:**

Minh Duc Do

Center for Molecular Biomedicine, University of Medicine and Pharmacy at Ho Chi Minh City, Vietnam

217 Hong Bang, District 5, Ho Chi Minh City, Vietnam, 700000

Email: ducminh@ump.edu.vn

Binh Quang Truong

Department of Internal Medicine, University of Medicine and Pharmacy at Ho Chi Minh City, 700000 Ho Chi Minh City, Vietnam

Cardiovascular Center, University Medical Center Ho Chi Minh City, 700000 Ho Chi Minh City, Vietnam

Email: binh.tq@umc.edu.vn

Abstract

Background: The pathogenesis and prognosis of patients with acute myocardial infarction (AMI) may be influenced by both genetic and environmental factors. Findings on the relationship of polymorphisms in genes encoding the renin-angiotensin-aldosterone system with coronary artery lesions and mortality in AMI patients are inconsistent. The aim of this study was to determine whether the *AGTR1* A1166C genetic polymorphism affects coronary artery lesions and 1-year mortality in post-AMI patients.

Methods: Patients with their first AMI admitted to Cho Ray Hospital, Vietnam, from January 2020 to August 2021 were enrolled in this prospective clinical study. All participants underwent invasive coronary angiography and were identified as having the genotypes of *AGTR1* A1166C by way of a polymerase chain reaction method. All patients were followed up for all-cause mortality 12 months after AMI. The association of the *AGTR1* A1166C polymorphism with coronary artery lesions and 1-year mortality were evaluated using logistic regression and Cox regression analysis, respectively.

Results: 531 AMI patients were recruited. The mean age was 63.9 ± 11.6 years, and 71.6% of the patients were male. There were no significant differences in the location and number of diseased coronary artery branches between the AA and AC+CC genotypes. The AC and CC genotypes were independently associated with ≥ 90% diameter stenosis of the left anterior descending (LAD) artery (odds ratio = 1.940; 95% confidence interval (CI): 1.059 - 3.552, $p = 0.032$). The difference in the 1-year all-cause mortality rate between patients with the AC and CC genotypes versus those with the AA genotype was not statistically significant (hazard ratio = 1.000, 95% CI: 0.429–2.328, $p = 1.000$).

Conclusions: The *AGTR1* A1166C genetic polymorphism is associated with very severe luminal stenosis of the LAD, but not with mortality in AMI patients.

Keywords

AGTR1 A1166C; genetic polymorphism; coronary artery lesion; mortality; acute myocardial infarction

Abbreviations

AMI, acute myocardial infarction; CI, confidence interval; CAD, coronary artery disease; AT1R, angiotensin II type I receptor RAAS, renin-angiotensin-aldosterone system; AT1, angiotensin II receptor type 1; LAD, left anterior descending artery; LVEF, left ventricular ejection fraction; DNA, Deoxyribonucleic acid; PCR, polymerase chain reaction; SD, standard deviation; IQR, interquartile range; OR, odds ratio; ACEI, angiotensin-converting enzyme inhibitor; ARB, angiotensin II receptor blocker; STEMI, ST-segment elevation myocardial infarction; eGFR, estimated glomerular filtration rate; LM, left main; LCx, left circumflex artery; RCA, right coronary artery; HR, hazard ratio.

1. Introduction

Coronary artery disease (CAD) is the predominant cause of death around the globe(1). Acute myocardial infarction (AMI) is the most severe clinical manifestation of CAD. The essential mechanism of AMI is the rupture or erosion of atherosclerotic plaques leading to thrombus formation causing complete or incomplete occlusion of epicardial coronary artery branches(2). In clinical practice, AMI is a common cardiovascular emergency and requires aggressive management. Although the survival status of patients with AMI has improved in recent decades, the burden of mortality and morbidity remains significant. The 1-year death rate in patients after AMI ranges from 10 to 12%(3).

Coronary artery lesions and mortality in AMI patients can be caused by both environmental and genetic factors. Many genetic markers associated with AMI have been identified, including genes encoding components of the renin-angiotensin-aldosterone system (RAAS). The *AGTR1* gene encoding the angiotensin II receptor type 1 (AT1) is located on the long arm of chromosome 3 (3q21-25), and A1166C is the most studied polymorphism(4). This gene variant is at the untranslated 3' region and has a base substitution of adenine with cytosine at nucleotide position 1166 of the mRNA sequence. In patients carrying the *AGTR1* A1166C genetic polymorphism, there is an increased expression of the AT1 receptor(5). Angiotensin II, the main effector in RAAS, acts on the AT1 receptor and causes vasoconstriction, aldosterone secretion, cell proliferation, vascular remodeling, endothelial dysfunction, and atherosclerosis(6,7).

The *AGTR1* A1166C genetic variant has been shown in previous studies to be associated with the risk of AMI. A meta-analysis comprising of 18 case-control studies demonstrated that the C allele is a risk factor for AMI(8). However, few studies have investigated the association between this genetic polymorphism and coronary artery lesions and mortality in patients with AMI. Kruzliak P *et al.* found that the CC genotype is associated with a higher risk of three-vessel stenosis and also a higher proportion of left anterior descending (LAD) artery infarction(9). In contrast, Araujo MA *et al.* did not detect a relationship between the *AGTR1* A1166C variant and coronary artery injuries when comparing the CC

genotype with AC and AA genotypes in AMI patients(10). However, *AGTR1* A1166C has been revealed in several studies to be a prognostic factor for mortality in patients with AMI(9,11,12), although other studies have not found any predictive value(13,14). Given the lack of data and the conflicting results between various studies, we conducted this study with the aim of investigating the effect of the *AGTR1* A1166C genetic variant on coronary artery lesion characteristics and mortality in Vietnamese patients with AMI.

2. Materials and Methods

2.1 Study population

In this prospective clinical study, we enrolled patients who were admitted to the Department of Cardiology and the Department of Interventional Cardiology at Cho Ray Hospital in Ho Chi Minh City, Vietnam, between January 2020 and August 2021. The inclusion criteria encompassed patients who were diagnosed with their first AMI according to the fourth universal definition(2) and who agreed to participate in the study. Exclusion criteria were: age below 18 years; history of AMI, percutaneous coronary intervention, and coronary artery bypass graft surgery; normal coronary angiogram; and loss of contact during the follow-up period.

The study was conducted in accordance with the principles of the Declaration of Helsinki and approved by the Ethics Committee of the University of Medicine and Pharmacy at Ho Chi Minh City. All participants provided written informed consent.

2.2 Study protocol

We collected data on participants' clinical characteristics, including age, sex, clinical type of AMI, and Killip class. Traditional cardiovascular risk factors were also collected, namely hypertension (according to ESC/ESH guidelines)(15), diabetes mellitus (according to ADA guidelines)(16), dyslipidemia (according to NCEP ATP-III guidelines)(17), obesity (according to the WHO classification for the Asian population)(18), smoking, and a family history of premature CAD. Laboratory parameters, such as Troponin I concentration, estimated glomerular filtration rate (eGFR), and left ventricular ejection fraction (LVEF) were also recorded. The LVEF of patients was assessed using Simpson's method for

echocardiography. All patients underwent invasive coronary angiography. The characteristics of coronary artery lesions were noted, i.e., the position of diseased coronary artery branches with ≥ 50% stenosis of the diameter, the number of diseased coronary artery branches, and very severe stenosis of the artery coronary artery branches (≥ 70% stenosis of the left main and ≥ 90% stenosis of the other epicardial coronary arteries). Two milliliters of venous blood were drawn from each patient for genotyping of the *AGTR1* A1166C polymorphism.

The patients were followed up during their hospital stay and up to 12 months from the date of admission. The primary outcome measured was all-cause mortality. Post-discharge follow-up was conducted through revisits and hospitalization at Cho Ray Hospital or telephone interviews.

2.3 Genotyping of AGTR1 A1166C

Genomic deoxyribonucleic acid (DNA) for all participants was extracted from peripheral leukocytes using the GeneJet™ Whole Blood Genomic DNA Purification Mini Kit (Thermo Fisher Scientific, Waltham, MA, USA) following the manufacturer's protocol. For each sample, *AGTR1* A1166C was genotyped by two separate polymerase chain reactions (PCR). Each reaction used three primers with similar melting temperatures to determine the presence of allele A or allele C based on the appearance of a 342-basepair DNA band on electrophoresis. All the PCRs were performed using a SimpliAmp™ Thermal Cycler (Thermo Fisher Scientific). The details of the PCR primers, components, and conditions are described in the Supplementary materials.

Twenty DNA samples were randomly chosen and directly sequenced using appropriate primers and conditions in an ABI 3500 sequencer (Thermo Fisher Scientific). The protocol for direct sequencing has been described previously(19–21). The DNA samples with an identified genotype were used as positive controls for PCR.

2.4 Statistical analysis

Qualitative variables were presented as frequencies and percentages. The Kolmogorov-Smirnov test was used to check the normal distribution of quantitative variables, which were expressed as mean with standard deviation (SD) if normally distributed or median

with interquartile range [IQR] if not normally distributed. Consistency with the Hardy-Weinberg equilibrium of genotypes was assessed using Chi-squared tests. Differences in the characteristics of coronary artery lesions between genotypes of the *AGTR1* A1166C genetic polymorphism were evaluated using Chi-squared or Fisher's exact tests. Factors affecting the degree of ≥ 90% stenosis of the LAD diameter were assessed by odds ratio (OR) and 95% CI in univariate and multivariable logistic regression analyses. Differences in the genotypes of the *AGTR1* A1166C polymorphism and other factors between those who survived and those who did not were compared using Chi-squared or Fisher's exact tests for qualitative variables and Student's t-test or the Mann-Whitney U test based on the data distribution. Factors associated with 1-year all-cause mortality in the univariate analysis were analyzed in a multivariable Cox regression model to identify independent prognostic factors.

Statistical analysis was performed using SPSS Statistics for Windows version 22.0 (IBM Corp., Armonk, NY, USA). A *p*-value of less than 0.05 was considered to be statistically significant.

3. Results

3.1 Characteristics of the study population

This study included 531 patients with their first AMI, most of whom were male (71.6%) (Table 1). The mean age of the participants was 63.9 ± 11.6 years. ST-segment elevation AMI was diagnosed in 63.3% of patients. Regarding the degree of acute heart failure, the proportion of patients with Killip class ≥ II was 23.5%. The prevalent diseased coronary artery was the LAD (89.1%). Three-vessel disease was predominant (40.7%) on coronary angiography. The majority of patients underwent coronary revascularization (94.7%) with fibrinolysis, percutaneous coronary intervention, or coronary bypass surgery. In addition, participants received standard medical therapy with aspirin (99.8%), P2Y12 inhibitor (100.0%), statin (98.5%), ACEI/ARB (90.4%), and beta-blocker (76.5%).

The genotypes of the *AGTR1* A1166C polymorphism in our study were not in agreement with the Hardy-Weinberg equilibrium ($p = 0.01$) (Table 2). The AA genotype was most

common in AMI patients (89.7%). In terms of traditional cardiovascular risk factors, dyslipidemia was the most frequent factor (89.6%), and a family history of premature CAD was not common (6.6%).

3.2 Association of AGTR1 A1166C *genetic polymorphism with coronary artery lesions in AMI patients*

There was no association between the *AGTR1* A1166C genetic variant and the location of diseased coronary artery branches, i.e., LM, LAD, LCx, and RCA (Table 3). No significant differences in the number of diseased coronary artery branches were found between patients carrying the AA genotype and those with the AC and CC genotype. However, the univariate analysis showed that patients with the AC and CC genotypes had a greater risk of ≥ 90% LAD stenosis than those with the AA genotype ($p = 0.028$). Univariate logistic regression analysis also showed that obesity and left ventricular ejection fraction were related to very severe stenosis of the LAD ($p = 0.022$ and $p < 0.001$, respectively). After adjusting for confounding factors in the multivariable logistic regression analysis, the AC and CC genotype was found to be independently associated with very severe stenosis of the LAD in AMI patients (OR = 1.940; 95% CI: 1.059 - 3.552; $p = 0.032$) (Table 4).

3.3 Association of the AGTR1 A1166C genetic polymorphism and other factors with 1-year all-cause mortality in AMI patients

There were 58 all-cause deaths during the 1-year follow-up (10.9%), including 52 patients with the AA genotype and 6 patients with the AC or CC genotype (Table 5). In univariate analysis, diabetes mellitus, age, Killip class ≥ II, Troponin I on admission, eGFR, LVEF, and ACEI/ARB therapy were found to be associated with 1-year all-cause mortality. The *AGTR1* A1166C genetic polymorphism had no effect on 1-year all-cause mortality, with HR = 1.000 (95% CI: 0.429 – 2.328; $p = 1.000$) in comparison between the AC+CC and AA genotypes. After adjusting for other factors in the multivariable Cox regression analysis, independent prognostic factors for 1-year all-cause mortality were Killip class ≥ II (HR = 2.375, 95% CI: 1.352 – 4.171, $p = 0.003$), admission Troponin I (HR = 1.001, 95% CI: 1.000 – 1.003, $p = 0.047$), and eGFR (HR = 0.985, 95% CI: 0.973 – 0.998, $p = 0.020$) (Table 6).

4. Discussion

The genotypes of the *AGTR1* A1166C polymorphism in our study were not in agreement with the Hardy-Weinberg equilibrium, possibly because our study population was a group of patients with AMI, not the entire population of case and control groups as in case-control studies. We did not select a control group because doing so was not necessary to answer the research question of whether the *AGTR1* A1166C variant is associated with coronary artery lesions and mortality in AMI patients. The CC genotype was the least common (1.1%) in our study population. This feature is consistent with other studies on different races, not just on Asian populations(9,10,22–24).

Our study showed that patients with the AC and CC genotypes had a higher rate of ≥ LAD 90% stenosis than AA genotype carriers. The study by Kruzliak P *et al*. demonstrated that acute coronary syndrome patients carrying the CC genotype in the Slovak Republic had a 4.08-fold higher risk of LAD infarction and a 3.87 times higher risk of three-vessel disease compared with those having the AA and AC genotypes(9). Nevertheless, the study by Araujo MA *et al.* found no differences in the number of diseased coronary vessels and morphological features of the atherosclerotic plaque among the *AGTR1* A1166C genotypes(10). The results of other studies are conflicting regarding the effect of *AGTR1* A1166C variants on coronary artery lesions due to differences in genotype distributions across countries and races, as well as the frequency of environmental factors affecting coronary atherosclerosis. In terms of pathogenesis, the AT1 receptor may be overexpressed in carriers of the *AGTR1* A1166C variant, leading to increased adverse effects of angiotensin II on coronary atherosclerosis. Angiotensin II promotes coronary artery injury possibly through various pathophysiological phenomena, such as modulation of the inflammatory response, stimulation of the production of various cytokines, such as IL-6, TNF-α, and COX-2, and promotion of the generation of reactive oxygen species, enhanced oxidative stress, decreased nitric oxide production, and increased endothelial dysfunction(3).

The *AGTR1* A1166C genetic polymorphism was not found to be associated with 1-year all-cause mortality in AMI patients in the present study. Similar results have been observed in

other studies(13,14). The CC genotype was not found to be associated with in-hospital mortality in the GEMIG (Genetics and Epidemiology of Acute Myocardial Infarction in the Greek Population) study in Greece(14). A study by Brscic E *et al.* involving young Italian patients with AMI concluded that the *AGTR1* A1166C polymorphism was not related to the composite end points of major cardiovascular events, including cardiovascular mortality, myocardial infarction, and revascularization procedures during the follow-up period of 46 ± 12 months(13).

In contrast, the *AGTR1* A1166C genetic polymorphism has been shown in several studies to be a predictor of mortality in AMI patients(9,11,12). Kruzliak P *et al.* found that the CC genotype is associated with a 6.48-fold increased risk of sudden cardiac death within 24 hours of emergency room admission in patients with acute coronary syndromes(9). After adjusting for the cause of death, the CC genotype was found in a large prospective study in France to be an independent predictor of post-AMI cardiovascular mortality during follow-up (median 2.5 years)(11). In addition, a study by Franco E *et al.* concluded that the AC genotype was associated with death, new myocardial infarction, and coronary revascularization in young Italian patients with AMI during the follow-up period of 9 ± 4 years(12).

The inconsistency in the results of various studies on the influence of the *AGTR1* A1166C genetic variant on mortality in AMI patients could be due to differences in the occurrence of the *AGRT1* A1166C genotypes across distinct ethnicities, countries, and geographic regions, as well as differences in study populations, study designs, and survival follow-up periods. In addition, ACEI and ARB have been shown in previous studies to improve mortality in patients after AMI(25–27). Therefore, the impact of ACEI and ARB might obscure the predictive value of the *AGTR1* A1166C polymorphism for mortality. The proportion of patients using ACEI/ARB in our study was very high (90.4%).

Our study had some limitations that should be taken into consideration. First, this study was conducted in only one center, so it might not be representative of the genotype characteristics of the *AGTR1* A1166C variant in Vietnam. Next, the pathophysiology of AMI includes the interaction of many factors and genes, so the *AGTR1* A1166C might not

fully explain the association of this polymorphism with coronary artery lesions and mortality in patients with AMI. Finally, the study was observational and thus did not intervene in treatment strategies and adherence to therapy in AMI patients.

5. Conclusions

The *AGTR1* A1166C genetic polymorphism is associated with very severe luminal stenosis of the LAD. Nevertheless, it is not associated with 1-year all-cause mortality in Vietnamese patients after AMI. These results contribute to the genetic assessment of coronary artery lesions, but further studies with larger sample sizes and longer follow-up time are needed to consider the prognostic role of the *AGTR1* A1166C variant in patients with AMI.

References:

1. GBD 2019 Diseases and Injuries Collaborators. Global burden of 369 diseases and injuries in 204 countries and territories, 1990-2019: a systematic analysis for the Global Burden of Disease Study 2019. Lancet Lond Engl. 2020 Oct 17;396(10258):1204–22.

2. Thygesen K, Alpert JS, Jaffe AS, Chaitman BR, Bax JJ, Morrow DA, et al. Fourth Universal Definition of Myocardial Infarction (2018). J Am Coll Cardiol. 2018 Oct 30;72(18):2231–64.

3. Laforgia PL, Auguadro C, Bronzato S, Durante A. The Reduction of Mortality in Acute Myocardial Infarction: From Bed Rest to Future Directions. Int J Prev Med. 2022;13:56.

4. Kariz S, Petrovic D. Genetic Markers of Myocardial Infarction. Clin Exp Cardiol. 2012;0(0):1–7.

5. Baudin B. Polymorphism in angiotensin II receptor genes and hypertension. Exp Physiol. 2005 May;90(3):277–82.

6. Reid IA. The renin-angiotensin system: physiology, pathophysiology, and pharmacology. Adv Physiol Educ. 1998 Dec 15;275(6):S236-245.

7. Poznyak AV, Bharadwaj D, Prasad G, Grechko AV, Sazonova MA, Orekhov AN. Renin-Angiotensin System in Pathogenesis of Atherosclerosis and Treatment of CVD. Int J Mol Sci. 2021 Jun 22;22(13):6702.

8. Feng X, Zheng BS, Shi JJ, Qian J, He W, Zhou HF. A systematic review and meta-analysis of the association between angiotensin II type 1 receptor A1166C gene polymorphism and myocardial infarction susceptibility. J Renin-Angiotensin-Aldosterone Syst JRAAS. 2014 Sep;15(3):307–15.

9. Kruzliak P, Kovacova G, Pechanova O, Balogh S. Association between angiotensin II type 1 receptor polymorphism and sudden cardiac death in myocardial infarction. Dis Markers. 2013;35(5):287–93.

10. Araújo MA, Goulart LR, Cordeiro ER, Gatti RR, Menezes BS, Lourenço C, et al. Genotypic interactions of renin-angiotensin system genes in myocardial infarction. Int J Cardiol. 2005 Aug 3;103(1):27–32.

11. Filippi-Codaccioni E, Morello R, Fradin S, Grollier G, Hamon M. Relation between the A1166C angiotensin II type 1 receptor gene polymorphism and cardiovascular outcomes after myocardial infarction. EuroIntervention J Eur Collab Work Group Interv Cardiol Eur Soc Cardiol. 2005 May;1(1):38–42.

12. Franco E, Palumbo L, Crobu F, Anselmino M, Frea S, Matullo G, et al. Renin-angiotensin-aldosterone system polymorphisms: a role or a hole in occurrence and long-term prognosis of acute myocardial infarction at young age. BMC Med Genet. 2007 May 22;8:27.

13. Brscic E, Bergerone S, Gagnor A, Colajanni E, Matullo G, Scaglione L, et al. Acute myocardial infarction in young adults: Prognostic role of angiotensin-converting enzyme, angiotensin II type I receptor, apolipoprotein E, endothelial constitutive nitric oxide synthase, and glycoprotein IIIa genetic polymorphisms at medium-term follow-up. Am Heart J. 2000 Jun 1;139(6):979–84.

14. Andrikopoulos GK, Tzeis SM, Needham EW, Richter DJ, Zairis MN, Gialafos EJ, et al. Lack of association between common polymorphisms in genes of the renin-angiotensin system and mortality after myocardial infarction. Cardiology. 2005;103(4):185–8.

15. Williams B, Mancia G, Spiering W, Agabiti Rosei E, Azizi M, Burnier M, et al. 2018 ESC/ESH Guidelines for the management of arterial hypertension. Eur Heart J. 2018 Sep 1;39(33):3021–104.

16. ElSayed NA, Aleppo G, Aroda VR, Bannuru RR, Brown FM, Bruemmer D, et al. 2. Classification and Diagnosis of Diabetes: Standards of Care in Diabetes-2023. Diabetes Care. 2023 Jan 1;46(Suppl 1):S19–40.

17. Expert Panel on Detection, Evaluation, and Treatment of High Blood Cholesterol in Adults. Executive Summary of The Third Report of The National Cholesterol Education Program (NCEP) Expert Panel on Detection, Evaluation, And Treatment of High Blood Cholesterol In Adults (Adult Treatment Panel III). JAMA. 2001 May 16;285(19):2486–97.

18. World Health Organization. Regional Office for the Western Pacific. The Asia-Pacific perspective : redefining obesity and its treatment [Internet]. Sydney: Health Communications Australia; 2000 [cited 2023 Aug 8]. 55 p. Available from: https://apps.who.int/iris/handle/10665/206936

19. Do MD, Pham DV, Le LP, Gia Le LH, Minh Tran LB, Dang Huynh MD, et al. Recurrent PROC and novel PROS1 mutations in Vietnamese patients diagnosed with idiopathic deep venous thrombosis. Int J Lab Hematol. 2021 Apr;43(2):266–72.

20. Do MD, Mai TP, Do AD, Nguyen QD, Le NH, Le LGH, et al. Risk factors for cutaneous reactions to allopurinol in Kinh Vietnamese: results from a case-control study. Arthritis Res Ther. 2020 Aug 3;22(1):182.

21. Kiet NC, Khuong LT, Minh DD, Quan NHM, Xinh PT, Trang NNC, et al. Spectrum of mutations in the RB1 gene in Vietnamese patients with retinoblastoma.

Mol Vis. 2019 Apr 4;25:215–21.

22. Bhoomi Reddy Pullareddy null, Baddela Muni Venkata Srikanth Babu null, Kolla Venkata Karunakar null, Yasovanthi J, Potham Sampath Kumar null, Sharath A, et al. Angiotensin II type 1 receptor gene polymorphism in myocardial infarction patients. J Renin-Angiotensin-Aldosterone Syst JRAAS. 2009 Sep;10(3):174–8.

23. Tiret L, Bonnardeaux A, Poirier O, Ricard S, Marques-Vidal P, Evans A, et al. Synergistic effects of angiotensin-converting enzyme and angiotensin-II type 1 receptor gene polymorphisms on risk of myocardial infarction. Lancet Lond Engl. 1994 Oct 1;344(8927):910–3.

24. Mehri S, Mahjoub S, Finsterer J, Zaroui A, Mechmeche R, Baudin B, et al. The CC genotype of the angiotensin II type I receptor gene independently associates with acute myocardial infarction in a Tunisian population. J Renin-Angiotensin-Aldosterone Syst JRAAS. 2011 Dec;12(4):595–600.

25. The Acute Infarction Ramipril Efficacy (AIRE) Study Investigators. Effect of ramipril on mortality and morbidity of survivors of acute myocardial infarction with clinical evidence of heart failure. The Lancet. 1993 Oct 2;342(8875):821–8.

26. Kim KH, Choi BG, Rha SW, Choi CU, Jeong MH. Impact of renin angiotensin system inhibitor on 3-year clinical outcomes in acute myocardial infarction patients with preserved left ventricular systolic function: a prospective cohort study from Korea Acute Myocardial Infarction Registry (KAMIR). BMC Cardiovasc Disord. 2021 May 21;21:251.

27. Pfeffer MA, Braunwald E, Moyé LA, Basta L, Brown EJ, Cuddy TE, et al. Effect of captopril on mortality and morbidity in patients with left ventricular dysfunction after myocardial infarction. Results of the survival and ventricular enlargement trial. The SAVE Investigators. N Engl J Med. 1992 Sep 3;327(10):669–77.

please delete (.)

Table 1. Baseline characteristics of the study population.

Variables	Characteristic (n = 531)
**Clinical and laboratory parameters**	
Age (years)	63.9 ± 11.6
Male	380 (71.6%)
STEMI	336 (63.3%)
Killip Class	
Class I	406 (76.5%)
Class II	44 (8.3%)
Class III	32 (6.0%)
Class IV	49 (9.2%)
Admission Troponin I (pg/mL)	15.1 (2.9 – 50.0)
eGFR (mL/min/1.73 m^2)	83.2 (64.3 – 94.5)
LVEF (%)	46.0 (39.0 – 53.0)
**Coronary artery lesions**	
Diseased LM	53 (10.0%)
Diseased LAD	473 (89.1%)
Diseased LCx	296 (55.7%)
Diseased RCA	380 (71.6%)
One-vessel disease	129 (24.3%)
Two-vessel disease	186 (35.0%)
Three-vessel disease	216 (40.7%)
≥ 70% LM stenosis	25 (4.7%)
≥ 90% LAD stenosis	254 (47.8%)
≥ 90% LCx stenosis	123 (23.2%)
≥ 90% RCA stenosis	234 (44.1%)
**Treatment**	
Coronary revascularization	503 (94.7%)

what about female?

Aspirin	530 (99.8%)
P2Y12 inhibitor	531 (100.0%)
Statin	523 (98.5%)
ACEI/ARB	480 (90.4%)
Beta-blocker	406 (76.5%)

Values are presented as the mean ± SD, number (%) or median (interquartile range). STEMI, ST-segment elevation myocardial infarction; eGFR, estimated glomerular filtration rate; LVEF, left ventricular ejection fraction; LM, left main; LAD, left anterior descending artery; LCx, left circumflex artery; RCA, right coronary artery; ACEI, angiotensin-converting enzyme inhibitor; ARB, angiotensin II receptor blocker.

Table 2. Genotypes of *AGTR1* A1166C polymorphism and traditional cardiovascular risk factors.

please delete (.)

Variables	Frequency (%) (n = 531)
**Genotypes of *AGTR1* A1166C polymorphism**	
AA	476 (89.7%)
AC	49 (9.2%)
CC	6 (1.1%)
**Traditional cardiovascular risk factors**	
Dyslipidemia	476 (89.6%)
Hypertension	435 (81.9%)
Smoking	224 (42.2%)
Diabetes mellitus	130 (24.5%)
Obesity	108 (20.3%)
Family history of premature CAD	35 (6.6%)

CAD, coronary artery disease.

Table 3. Association between *AGTR1* A1166C genetic polymorphism and coronary artery lesions. please delete (.)

Variables	AA (n = 476)	AC+CC (n = 55)	*p*-value
Diseased LM	46 (9.7%)	7 (12.7%)	0.473
Diseased LAD	422 (88.7%)	51 (92.7%)	0.359
Diseased LCx	264 (55.5%)	32 (58.2%)	0.701
Diseased RCA	342 (71.8%)	38 (69.1%)	0.668
One-vessel disease	116 (24.4%)	13 (23.6%)	0.904
Two-vessel disease	168 (35.3%)	18 (32.7%)	0.706
Three-vessel disease	192 (40.3%)	24 (43.6%)	0.637
≥ 70% LM stenosis	21 (4.4%)	4 (7.3%)	0.314
≥ 90% LAD stenosis	220 (46.2%)	34 (61.8%)	0.028
≥ 90% LCx stenosis	110 (23.1%)	13 (23.6%)	0.930
≥ 90% RCA stenosis	215 (45.2%)	19 (34.5%)	0.133

LM, left main; LAD, left anterior descending artery; LCx, left circumflex artery; RCA, right coronary artery.

Table 4. Effect of the *AGTR1* A1166C genetic polymorphism and other factors on very severe stenosis of the left anterior descending artery.

please delete (.)

Variables	Unadjusted OR (95% CI)	*p*-value	Adjusted OR (95% CI)	*p*-value
AC+CC vs. AA	1.884 (1.062 – 3.342)	0.030	1.940 (1.059 – 3.552)	0.032
Dyslipidemia	1.025 (0.586 – 1.794)	0.930		
Hypertension	1.048 (0.673 – 1.632)	0.835		
Smoking	0.853 (0.604 – 1.204)	0.365		
Diabetes mellitus	0.843 (0.566 – 1.254)	0.398		
Obesity	0.603 (0.391 – 0.930)	0.022	0.647 (0.409 – 1.021)	0.062
Family history of premature CAD	1.167 (0.588 – 2.316)	0.660		
Age (years)	1.014 (0.999 – 1.029)	0.073		
Male	0.870 (0.596 – 1.268)	0.468		
STEMI	1.353 (0.949 – 1.930)	0.095		
Killip Class ≥ II	1.075 (0.720 – 1.603)	0.724		
Admission Troponin I (pg/mL)	1.000 (0.997 – 1.002)	0.798		
eGFR (mL/min/1.73 m^2)	0.999 (0.992 – 1.006)	0.798		
LVEF	0.940 (0.923 – 0.957)	<0.001	0.940 (0.924 – 0.957)	<0.001

How its calculated, despite you have only one group?

CAD, coronary artery disease; STEMI, ST-segment elevation myocardial infarction; eGFR, estimated glomerular filtration rate; LVEF, left ventricular ejection fraction.

Table 5. Association of the *AGTR1* A1166C genetic polymorphism and other factors with 1-year all-cause mortality in AMI patients. please delete (.)

Variables	Death (n = 58)	Survivor (n = 473)	*p*-value
**Genotypes of *AGTR1* A1166C polymorphism**			
AA	52 (89.7%)	424 (89.6%)	0.997
AC+CC	6 (10.3%)	49 (10.4%)	
**Traditional cardiovascular risk factors**			
Dyslipidemia	52 (89.7%)	424 (89.6%)	0.997
Hypertension	48 (82.8%)	387 (81.8%)	0.861
Smoking	22 (37.9%)	202 (42.7%)	0.487
Diabetes mellitus	22 (37.9%)	108 (22.8%)	0.012
Obesity	10 (17.2%)	98 (20.7%)	0.535
Family history of premature CAD	4 (6.9%)	31 (6.6%)	0.785
**Clinical and laboratory factors**			
Age (years)	67.5±10.5	63.5±11.7	0.013
Male	36 (62.1%)	344 (72.7%)	0.089
STEMI	38 (65.5%)	298 (63.0%)	0.708
Killip Class ≥ II	29 (50.0%)	97 (20.5%)	<0.001
Admission Troponin I (pg/mL)	37.5 (3.26 – 50.0)	13.9 (2.7 – 50.0)	0.029
eGFR (mL/min/1.73 m^2)	62.1 (40.5 – 86.7)	84.6 (67.7 – 95.0)	<0.001
LVEF (%)	40.5 (31.5 – 50.0)	47.0 (40.0 – 53.0)	0.002
**Coronary artery lesions**			
Diseased LM	7 (12.1%)	46 (9.7%)	0.574

Diseased LAD	53 (91.4%)	420 (88.8%)	0.551
Diseased LCx	32 (55.2%)	264 (55.8%)	0.926
Diseased RCA	42 (72.4%)	338 (71.5%)	0.879
One-vessel disease	12 (20.7%)	117 (24.7%)	0.498
Two-vessel disease	23 (39.7%)	163 (34.5%)	0.434
Three-vessel disease	23 (29.7%)	193 (40.8%)	0.867
≥ 70% LM stenosis	5 (8.6%)	20 (4.2%)	0.177
≥ 90% LAD stenosis	30 (51.7%)	224 (47.4%)	0.530
≥ 90% LCx stenosis	18 (31.0%)	105 (22.2%)	0.132
≥ 90% RCA stenosis	27 (46.6%)	207 (43.8%)	0.686
**Treatment**			
Coronary revascularization	53 (91.4%)	450 (95.1%)	0.216
Aspirin	57 (98.3%)	473 (100.0%)	0.109
P2Y12 inhibitor	58 (100.0%)	473 (100.0%)	-
Statin	57 (98.3%)	466 (98.5%)	0.606
ACEI/ARB	47 (81.0%)	433 (91.5%)	0.010
Beta-blocker	43 (74.1%)	363 (76.7%)	0.659

Values are presented as number (%) or median (interquartile range). CAD, coronary artery disease; STEMI, ST-segment elevation myocardial infarction; eGFR, estimated glomerular filtration rate; LVEF, left ventricular ejection fraction; LM, left main; LAD, left anterior descending artery; LCx, left circumflex artery; RCA, right coronary artery; ACEI, angiotensin-converting enzyme inhibitor; ARB, angiotensin II receptor blocker.

Table 6. Factors associated with 1-year all-cause mortality in multivariable Cox regression analysis.

please delete (.)

Variables	HR	95% CI	*p*-value
Diabetes mellitus	1.338	0.761 – 2.352	0.312
Age (years)	1.013	0.988 – 1.038	0.309
Killip Class ≥ II	2.375	1.352 – 4.171	0.003
Admission Troponin I (pg/mL)	1.001	1.000 – 1.003	0.047
eGFR (mL/min/1.73 m^2)	0.985	0.973 – 0.998	0.020
LVEF (%)	0.977	0.954 – 1.002	0.068
ACEI/ARB	0.662	0.326 – 1.343	0.253

eGFR, estimated glomerular filtration rate; LVEF, left ventricular ejection fraction; ACEI, angiotensin-converting enzyme inhibitor; ARB, angiotensin II receptor blocker.

Availability of Data and Materials

The datasets generated and analyzed during the current study are not publicly available due to patient privacy but are available from the corresponding author on reasonable request.

Author Contributions

DT, TT, SH, MD, and BT designed the research study. DT, SH, and LL recruited the participants for the study. LL, MD, and DT performed the genotyping procedure. DT, TT, SH, MD, and BT analyzed the data. DT, MD, and TT wrote the manuscript. All authors contributed to editorial changes in the manuscript. All authors read and approved the final manuscript.

Ethics Approval and Consent to Participate

The study was approved by the local ethics committee of University of Medicine and Pharmacy at Ho Chi Minh City (Protocol No. 550/UMP-BOARD). All patients gave their informed consent to participate in this study.

Acknowledgment

Not applicable.

Funding

This study was supported partially by the University of Medicine and Pharmacy at Ho Chi Minh City, Vietnam. Duy Cong Tran was funded by the Master, PhD Scholarship Program of Vingroup Innovation Foundation (VINIF), code VINIF.2022.TS027.

Conflict of Interest

The authors report no relationships that could be construed as a conflict of interest.

Click here to access/download
Supporting Information
Supplemental material.docx
