# Supplementary material for: Effect of AGTR1 A1166C genetic polymorphism on coronary artery lesions and mortality in patients with acute myocardial infarction
Source: PLoS One. 2024 Apr 18;19(4):e0300273. doi: 10.1371/journal.pone.0300273 (PMC11026145; doi:10.1371/journal.pone.0300273)
Supplement: Attachment — Submitted filename: Response to Reviewers.docx [file pone.0300273.s005.docx]

**RESPONSE TO REVIEWERS**

**Reviewer #1:**

**1. I have minor correction fixed on the manuscript.**

*Thank you for your careful reading and correction. We corrected several points according to your suggestion on the manuscript.*

**2. I have a question related to the table 4.**

*We calculated the odds ratio of the association between the AGTR1 A1166C genetic polymorphism and very severe stenosis of the left anterior descending artery (LAD) among acute myocardial infarction patients based on the number of participants in groups listed in the following table.*

| ***Group*** | ***Very severe stenosis of LAD*** | ***No severe stenosis of LAD*** | ***Total*** |
| --- | --- | --- | --- |
| ***AC+CC*** | *34* | *21* | *55* |
| ***AA*** | *220* | *256* | *476* |
| ***Total*** | *254* | *277* | *531* |

**Reviewer #2:** **The manuscript addresses an important clinical question related to the genetics of AMI and its associated outcomes. However, the manuscript contains fundamental errors that cannot be rectified through author revisions. Specifically, the number of specific subjects is insufficient for this study.**

*Thank you for your comment. The initial sample size was calculated to estimate the difference in the survival rates among different genotypes of AGTR1 A1166C polymorphism. A minimum of 33 deaths or 413 AMI patients were needed to have a statistical power of at least 80% with a 95% confidence interval to detect a hazard ratio of 2 or more. In reality, there were 531 AMI patients enrolled and 58 all-cause deaths during the 1-year follow-up in our study.*

**Reviewer #3:**

*We would like to thank you Reviewer 3 for the detailed comments for our manuscript. We would like to address all the comments point by point:*

**First comment:** **The study design seems bizarre and not sound.
The design and analysis of genetic polymorphisms studies generally involve relating a particular disease or health outcome to a particular exposure or genetic trait, while assessing the presence of systematic error, controlling random error and assessing effect modification (interaction) with other exposures or traits.**

**To achieve these requirements, there should be two distinct groups to compare -usually -normal controls are used in this type of studies as comparative group.
Also, the authors stated in the discussion that “they did not select a control group because doing so was not necessary to answer the research question of whether the AGTR1 A1166C variant is associated with coronary artery lesions and mortality in AMI patients”. How they proved that this variant is associated with the lesions and mortality without comparing it with normal population?
What if the variant prevalence in normal population is similar to their current results?**

*Despite the diverse evidence of genetic variants in the renin-angiotensin-aldosterone system in primary prevention setttings of cardiovascular diseases such as acute myocardial infarction, whether it affects coronary artery lesions and mortality in patients diagnosed with AMI in the secondary prevention setting has not been well studied and remains controversial.* *A case-control study design is needed to determine the association between AGTR1 A1166C genetic polymorphism and the risk of AMI in primary prevention settings. Nevertheless, we aimed to investigate the association of AGTR1 A1166C genotypes with coronary artery lesions and mortality in patients with AMI in the secondary prevention setting. Therefore, the control group might not be needed to answer the research question in our study. Similarly, several previous studies by Tokunaga S et al (2001) (****ref: doi:*** *10.2143/AC.56.6.2005698), Palmer BR et al (2003) (****ref: doi:*** *10.1016/s0735-1097(02)02927-3), Goldenberg I et al (2006)* ***(ref: doi:*** [10.1161/01.HYP.0000239204.41079.6b](https://doi.org/10.1161/01.hyp.0000239204.41079.6b)), *Hara M et al,* [*OACIS Investigators*](https://pubmed.ncbi.nlm.nih.gov/?term=OACIS+Investigators%5BCorporate+Author%5D) *(2014) (****ref: doi:*** [*10.1536/ihj.13-288*](https://doi.org/10.1536/ihj.13-288)*),* Martínez-Quintana E *et al (2014) (****ref: doi:*** [10.5603/CJ.a2013.0107](https://doi.org/10.5603/cj.a2013.0107)) *and Moorthy N et al (2021) (****ref: doi:*** *10.1016/j.ijcha.2020.100701)* *also did not recruit a control group. Therefore, we did not select a control group, but compared groups with different genotypes of AGTR1 A1166C polymorphism among AMI patients.*

**Second comment:** How the author calculated the Hardy-Weinberg equilibrium without having two groups (cases and controls)?

*The Hardy-Weinberg equilibrium can be calculated in either cases or controls. Thus, the Hardy-Weinberg equilibrium of genotypes of the AGTR1 A1166C polymorphism in AMI patients was calculated using Chi-squared test in SPSS Statistics for Windows version 22.0 (IBM Corp., Armonk, NY, USA) based on observed and expected numbers of participants in different genotype groups.*

| **Genotype group** | **Observed number of participiants** | **Expected number of participiants** |
| --- | --- | --- |
| AA | 476 | 472 |
| AC | 49 | 57 |
| CC | 6 | 2 |

*AGTR1 A1166C genotypes in our study population were not at Hardy-Weinberg equilibrium (P < 0.05).* *This could be explained by the fact that our study included only AMI patients. Deviation from Hardy-Weinberg equilibrium can also be caused by selection bias or error in genotyping. Nevertheless, selection bias was tightly controlled by accurately selecting AMI subjects who satisfied the sampling criteria through careful history taking, thorough clinical examination, and performing all necessary tests to diagnose AMI definitely. In addition, the genetic testing in this study was performed according to precise and rigorous procedures.*

**Third comment:** On what basis they used genotypes AC+CC vs. AA in most of their comparisons?
 *As the number of paticipants with AGTR1 CC genotype was rare and alen C was consider “the risk group” according to previous studies in the literature, we used the recessive genetic model to compare AC+CC vsersus AA in terms of coronary artery lesions and mortality in AMI patients.*

**Fourth comment:** There are many grammatical and prepositions errors throughout the manuscript, and it needs major English Language revision.

*We revised grammatical and prepositions errors in our manuscript.*

Once again, we would like to thank Reviewer 3 for your time in reviewing our manuscript.

REFERENCE:

1. Tokunaga S, Tsuji H, Nishiue T, et al. Lower mortality in patients with the DD genotype of the angiotensin-converting enzyme gene after acute myocardial infarction. *Acta Cardiol.* 2001;56:351-5. doi: 10.2143/AC.56.6.2005698.
2. Palmer BR, Pilbrow AP, Yandle TG, et al. Angiotensin-converting enzyme gene polymorphism interacts with left ventricular ejection fraction and brain natriuretic peptide levels to predict mortality after myocardial infarction. *J Am Coll Cardiol*. 2003;41(50):729-36. doi: 10.1016/s0735-1097(02)02927-3.
3. Goldenberg I, Moss AJ, Ryan D, et al. Polymorphism in the angiotensinogen gene, hypertension, and ethnic differences in the risk of recurrent coronary events. *Hypertension*. 2006;48:693-699. doi: [10.1161/01.HYP.0000239204.41079.6b](https://doi.org/10.1161/01.hyp.0000239204.41079.6b).
4. Hara M, Sakata Y, Nakatani D, et al. Renin-angiotensin-aldosterone system polymorphism and 5-year mortality in survivors of acute myocardial infarction. *Int Heart J*. 2014;55:190-196. doi: [10.1536/ihj.13-288](https://doi.org/10.1536/ihj.13-288). doi: [10.5603/CJ.a2013.0107](https://doi.org/10.5603/cj.a2013.0107).
5. Martinez-Quintana E, Chirino R, Nieto-Lago V, et al. Prognostic value of *ACE* I/D, *AT1R* A1166C, *PAI-I* 4G/5G and *GPIIIa* a1/a2 polymorphisms in myocardial infarction. *Cardiol J*. 2014;21(3):229-237.
6. Moorthy N, Ramegowda KS, Jain S, et al. Role of Angiotensin-Converting Enzyme (ACE) gene polymorphism and ACE activity in predicting outcome after acute myocardial infarction. *Int J Cardiol Heart Vasc*. 2021;32:100701. doi: 10.1016/j.ijcha.2020.100701.
